# Mitochondrial STAT5A promotes metabolic remodeling and the Warburg effect by inactivating the pyruvate dehydrogenase complex

**DOI:** 10.1038/s41419-021-03908-0

**Published:** 2021-06-19

**Authors:** Liang Zhang, Jianong Zhang, Yan Liu, Pingzhao Zhang, Ji Nie, Rui Zhao, Qin Shi, Huiru Sun, Dongyue Jiao, Yingji Chen, Xiaying Zhao, Yan Huang, Yao Li, Jian-Yuan Zhao, Wei Xu, Shi-Min Zhao, Chenji Wang

**Affiliations:** 1 Obstetrics & Gynecology Hospital of Fudan University, State Key Laboratory of Genetic Engineering, MOE Engineering Research Center of Gene Technology, Key Laboratory of Reproduction Regulation of NPFPC (SIPPR, IRD), School of Life Sciences, Fudan University, 200438 Shanghai, China; 2 Institute of metabolism and integrative biology (IMIB), School of Life Sciences, Fudan University, 200438 Shanghai, China; 3 Fudan University Shanghai Cancer Center and Department of Pathology, School of Basic Medical Sciences, Shanghai Medical College, Fudan University, 200032 Shanghai, China; 4 State Key Laboratory of Genetic Engineering, School of Life Sciences, Fudan University, 200438 Shanghai, China; 5 Shanghai Fifth People’s Hospital and Institutes of Biomedical Sciences, Fudan University, 20032 Shanghai, China

**Keywords:** Cancer metabolism, Cell growth

## Abstract

Signal transducer and activator 5a (STAT5A) is a classical transcription factor that plays pivotal roles in various biological processes, including tumor initiation and progression. A fraction of STAT5A is localized in the mitochondria, but the biological functions of mitochondrial STAT5A remain obscure. Here, we show that STAT5A interacts with pyruvate dehydrogenase complex (PDC), a mitochondrial gatekeeper enzyme connecting two key metabolic pathways, glycolysis and the tricarboxylic acid cycle. Mitochondrial STAT5A disrupts PDC integrity, thereby inhibiting PDC activity and remodeling cellular glycolysis and oxidative phosphorylation. Mitochondrial translocation of STAT5A is increased under hypoxic conditions. This strengthens the Warburg effect in cancer cells and promotes in vitro cell growth under hypoxia and in vivo tumor growth. Our findings indicate distinct pro-oncogenic roles of STAT5A in energy metabolism, which is different from its classical function as a transcription factor.

## Introduction

Cells constantly consume glucose and oxygen for energy production. Normal cells rely primarily on mitochondrial oxidative phosphorylation (OXPHOS) to produce ATP [1, 2]. However, under hypoxia, glucose is converted to lactate through glycolysis to produce ATP [3]. The abnormal dependence on glycolysis as the main source of ATP, even in the presence of oxygen is seen in many cancer cells and is commonly called the “Warburg effect” [4]. However, the mechanisms by which increased reliance on glycolysis promotes cancer initiation and progression are not fully understood [5].

The pyruvate dehydrogenase complex (PDC) is a large nuclear-encoded multienzyme complex that is localized in the mitochondrial matrix [6]. It catalyzes the irreversible decarboxylation of pyruvate, the end product of glycolysis, to acetyl coenzyme A (acetyl-CoA), which feeds the tricarboxylic acid (TCA) cycle, leading to the formation of citrate. In this way, PDC is a gatekeeper enzyme connecting two key metabolic pathways of glycolysis and the TCA cycle [7–9]. PDC is organized around a 60-meric dodecahedral core with four major components: pyruvate dehydrogenase (PDH) (PDHA1/PDHB, E1), dihydrolipoyl transacetylase (DLAT, E2), dihydrolipoamide dehydrogenase (DLD, E3), and E3-binding protein (E3BP) [10, 11]. PDC plays central roles in the regulation of cellular energy and the supply of intermediates for biosynthesis, its activity is dynamically regulated by various cellular inputs [7]. The best-known regulatory model of PDC involves the reversible phosphorylation/dephosphorylation of PDHA1 catalyzed by specific mitochondrial pyruvate dehydrogenase kinases (PDK1–4) and pyruvate dehydrogenase phosphatases (PDP1–2), respectively. Phosphorylation of three serine residues (Ser232, Ser293, and Ser300) of PDHA1 inactivates PDC while dephosphorylation activates PDC [12–14]. Furthermore, recent studies demonstrated that PDC activity can be regulated by acetylation, ubiquitination, and tyrosine phosphorylation, which add additional levels of complexity to the regulation of PDC activity [14–16].

STAT5A and its closely related paralog, STAT5B, are part of the Janus kinase (JAK)/STAT signaling pathway, which can be activated by cytokines and growth factors, and which participates in a variety of essential functions, such as cell proliferation, differentiation, apoptosis, survival, and senescence [17–19]. Upon activation, STAT5A is tyrosine phosphorylated by upstream kinases, including JAK family members, and undergoes conformational changes to form active, parallel dimers that translocate to the nucleus. There, it promotes the transcription of target genes and alters the enhancer-promoter landscape of chromatin [20]. STAT5A/B is frequently overexpressed in various cancers [21]. The recent discovery of STAT5A/B mutations in cancers and functional studies indicated that STAT5A/B mutants are oncogenic [22]. Therefore, pharmacological interventions of STAT5/B are potential therapeutic strategies for malignant cancers [21, 23].

STATs have been studied extensively and are involved in almost all biochemical pathways [24, 25]. Until recently, however, they were thought to exert these effects solely as nuclear transcription factors. Accumulating evidence indicated that all STAT family members can localize to mitochondria [26]. For example, recent studies revealed a nonclassical function of mitochondrial STAT3 as a modulator of mitochondrial respiration and reactive oxygen species (ROS) [27–29]. Although an early report showed that STAT5A underwent cytokine-stimulated mitochondrial translocation and interaction with DLAT, the functional impacts of mitochondrial STAT5A on PDC activity and energy metabolism are not well-established [30]. To address these issues, we used multiple biochemical and cellular approaches to demonstrate that mitochondrial STAT5A acts as an inhibitor of PDC activity, thereby promoting metabolic remodeling and supporting tumor growth.

## Results

### Identification of STAT5A as a PDC interactor

**Fig. 1:**
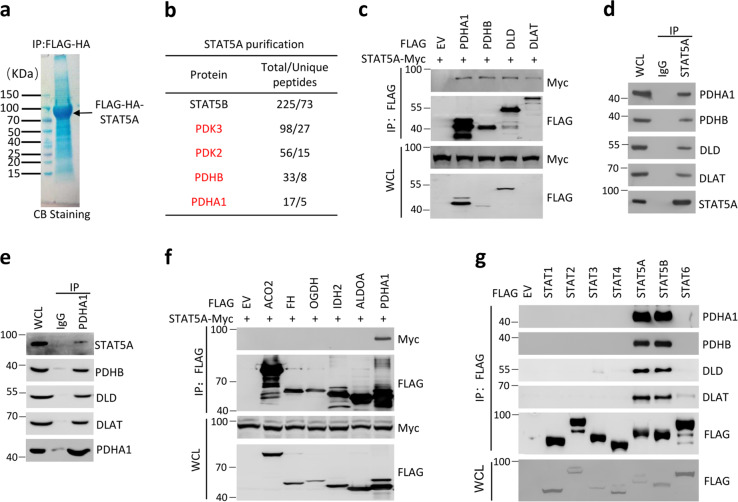
STAT5A interacts with PDC in 293T cells. **a**, **b** Tandem affinity purification of STAT5A-containing protein complexes was conducted using 293T cells stably expressing FLAG-HA-STAT5A. Associated proteins were separated by SDS-PAGE and visualized by Coomassie Blue (CB) staining (**a**). The numbers of total/unique peptides identified by mass spectrometry are shown in the table (**b**). **c** 293T cells were co-transfected with indicated plasmids. Whole-cell lysates (WCL) were immunoprecipitated with anti-FLAG antibody, and the immunoprecipitated proteins were immunoblotted with antibodies specific to the Myc or the FLAG. The total proteins used for immunoprecipitation were 4–5 mg. The proteins used for Immunoblot were 20–30 μg per lane. **d** Co-immunoprecipitation of STAT5A with PDC from 293T cells. WCL (lane 1) or immunoprecipitates generated with the STAT5A antibody (lane 3) or a control IgG (lane 2) were immunoblotted with indicated antibodies. The total proteins used for immunoprecipitation were 10–12 mg. The proteins used for Immunoblot were 45–60 μg per lane. **e** Co-immunoprecipitation of PDHA1 with STAT5A from 293T cells. WCL (lane 1) and immunoprecipitates generated with the PDHA1 antibody (lane 3) or a control IgG (lane 2) were immunoblotted with indicated antibodies. The total proteins used for immunoprecipitation were 10–12 mg. The proteins used for immunoblot were 45–60 μg per lane. **f** 293T cells were co-transfected with indicated expression vectors. WCL were immunoprecipitated with anti-FLAG antibody, and the immunoprecipitated proteins were immunoblotted with antibody specific to the Myc (for STAT5A; upper panel) or the FLAG (lower panel). The total proteins used for immunoprecipitation were 4–5 mg. The proteins used for immunoblot were 20–30 μg per lane. **g** 293T cells were transfected with expression vectors encoding FLAG-STATs. WCL were immunoprecipitated with anti-FLAG antibody, and the immunoprecipitated proteins were immunoblotted with indicated antibodies. The total proteins used for immunoprecipitation were 4–5 mg. The proteins used for Immunoblot were 20–30 μg per lane.

To identify the transcription-independent oncogenic functions of STAT5A, we isolated STAT5A protein complexes from 293T cells stably expressing FLAG-HA-STAT5A through tandem affinity purification methods and determined the proteins present in the complexes using mass spectrometry (TAP-MS) (Fig. 1a, b). Verification of the efficiency of this approach was confirmed by detecting STAT5B, a close paralog of STAT5A, in the complexes. This was not surprising because STAT5A and STAT5B form stable heterodimeric complexes [21]. Furthermore, the PDC subunits (PDHA1, PDHB, and DLAT) and their regulatory kinases (PDK2 and PDK3) were present in the complexes. These results are largely consistent with data from a recent large-scale protein interactome mapping study of 293T cells using STAT5A-HA as bait [31]. We coexpressed Myc–STAT5A and FLAG-tagged PDC subunits, including PDHA1, PHDB, DLD, and DLAT in 293T cells, and performed co-immunoprecipitation (co-IP) analyses with an anti-FLAG antibody. As shown in Fig. 1c, Myc–STAT5A was successfully co-immunoprecipitated by FLAG-tagged PDC subunits, indicating that an interaction occurs between STAT5A and PDC. Co-IP assays with an anti-STAT5A antibody demonstrated that endogenous STAT5A interacted with multiple PDC subunits, including PDHA1, PHDB, DLD, and DLAT in 293T, HeLa (a human cervical cancer cell line), MIHA (a human hepatocyte), and primary mouse hepatocytes (Fig. 1d and Supplementary Fig. 1a–c). Reciprocally, PDHA1 was able to immunoprecipitate endogenous STAT5A and other PDC subunits (Fig. 1e). By contrast, no interaction was observed between STAT5A and other metabolic enzymes of the TCA cycle examined (ACO2, FH, OGDH, IDH2, and ALDOA) (Fig. 1f). Finally, we demonstrated that STAT5B displays a similar binding affinity for PDC as STAT5A (Fig. 1g). This result is not surprising because STAT5A and STAT5B share 93% protein sequence identity and most of the biological functions of the two proteins overlap [21]. By contrast, no interactions were observed between PDC and other STAT family members (Fig. 1g).

Taken together, these results indicate that a specific interaction between STAT5A and PDC occurs in human and mouse cells.

### STAT5A interacts predominantly with PDC in the mitochondria

**Fig. 2:**
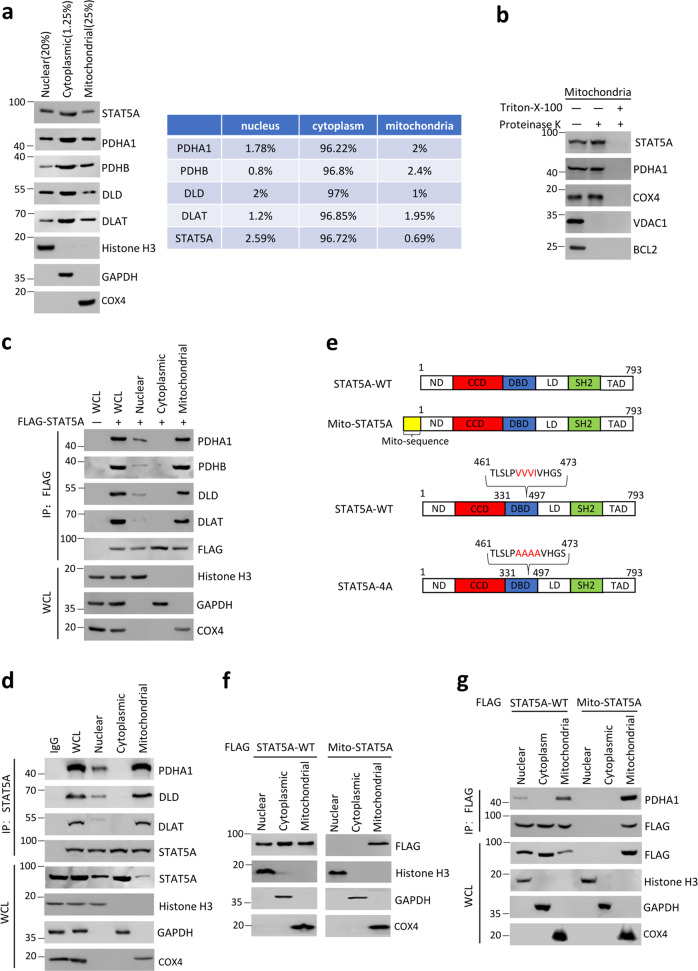
The mitochondria is a main cellular compartment that STAT5A–PDC interaction occurs. **a** The cytoplasmic, mitochondrial, and nuclear fractions from HeLa cells were prepared as described in the “Materials and methods” section. STAT5A and PDC subunit (PDHA1, PDHB, DLD, and DLAT) in the three fractions were detected by immunoblot. Histone H3 (nucleus), GAPDH (cytoplasm), and COX4 (mitochondria) were used as subcellular fraction markers. The total proteins used for subcellular fractionation were 4–5 mg. **b** Mitochondria were fractionated from HeLa cells and incubated with (lanes 2 and 3) or without (lane 1) proteinase K. To disrupt mitochondrial integrity, Triton X-100 was added in the digestion buffer (lane 3). The indicated proteins in the reactions were detected by Immunoblot. The total proteins used for isolation of mitochondria were 4–5 mg. **c** The nuclear, cytoplasmic, and mitochondrial fractions from HeLa cells transfected with expression vectors encoding EV or FLAG-STAT5A. WCL were immunoprecipitated with anti-FLAG antibody, and the immunoprecipitated proteins were immunoblotted with indicated antibodies. **d** The nuclear, cytoplasmic, and mitochondrial fractions from HeLa cells were subjected to immunoprecipitation with anti-STAT5A antibody, and the immunoprecipitated proteins were immunoblotted with indicated antibodies. **e** Schematic diagram of STAT5A-WT, Mito-STAT5A, and STAT5A-4A mutant. **f** The nuclear, cytoplasmic, and mitochondrial fractions from HeLa cells transfected with indicated expression vectors. The fractionated proteins were immunoblotted with indicated antibodies. **g** HeLa cells were transfected with expression vectors encoding FLAG-STAT5A-WT or FLAG-Mito-STAT5A.WCL were immunoprecipitated with anti-FLAG antibody, and the immunoprecipitated proteins were immunoblotted with indicated antibodies.

To define the subcellular compartments in which the STAT5A–PDC interaction occurs, we separated the nuclear, mitochondrial, and cytoplasmic fractions of HeLa cells using density-gradient centrifugation methods. The relative amounts of STAT5A in three fractions were detected by Immunoblot. An estimation based on band intensity quantification and fraction amounts indicated that a small amount (0.69%) of STAT5A is localized in mitochondria, while the cytoplasmic and nuclear fractions of STAT5A are 96.72% and 2.59%, respectively. PDC subunits were localized predominantly in the cytoplasm with a small amount localized in the nucleus and mitochondria. By contrast, COX4—a mitochondrial protein marker—was exclusively present in the mitochondrial fractions (Fig. 2a). To exclude the possibility that STAT5A was adhered to the outer membrane of mitochondria in a potentially non-specific manner, we incubated the isolated mitochondrial fractions with proteinase K in the presence or absence of the detergent Triton X-100. Proteins associated with the outer mitochondrial membrane are expected to be protease-sensitive, whereas internal proteins are degraded only after disruption of the mitochondrial membrane by Triton X-100 (ref. [28]). Immunoblot analyses showed that STAT5A, PDHA1, and COX4 (an inner mitochondrial membrane protein) were resistant to proteinase K, whereas BCL2 and VDAC1, both of which are localized on the outer membrane of mitochondria, were degraded. When proteinase K was added to the mitochondria fractions in the presence of Triton X-100, all the tested proteins were degraded (Fig. 2b). These results indicate that STAT5A is probably localized in the internal compartment, but not on the outer membrane of the mitochondria.

After determining that STAT5A and PDC are present in all three fractions, we investigated in which cell compartments where STAT5A and PDC interact. The same amounts of FLAG-STAT5A in the three separated fractions were subjected to co-IP and immunoblot analyses. Mitochondrial STAT5A immunoprecipitated much more PDC than nuclear STAT5A, while no interaction between cytoplasmic STAT5A and PDC was observed (Fig. 2c). Similar results were obtained when anti-STAT5A antibody was used for endogenous co-IP assays (Fig. 2d). To further confirm that STAT5A–PDC interaction occurs in mitochondria, we constructed a mito-STAT5A plasmid artificially targeted to mitochondria. A mitochondrial targeting sequence derived from NDUFV2 (an integral mitochondrial protein) was added to the N-terminus sequence of STAT5A (Fig. 2e) [32]. In contrast to STAT5A-WT, mito-STAT5A was exclusively localized to mitochondria (Fig. 2f). Co-IP assays demonstrated that mito-STAT5A specifically interacted with the mitochondrial PDC, but not the nuclear PDC (Fig. 2g).

In a number of cell types, STAT3 are able to localize to mitochondria in a manner that is dependent on phosphorylation of Ser727 in its TAD domain [28, 29]. We investigated whether the mitochondrial translocation of STAT5A is related to its phosphorylation status. We chose several reported STAT5A phosphorylation sites, including Ser127, Ser128, Tyr694, Thr757, and Ser780 (refs. [33–36]). We generated a panel of non-phosphorylatable SA (Ser to Ala) and phosphomimetic SD (Ser to Asp) mutants of STAT5A and tested their abilities to interact with PDC and localize to mitochondria. As shown in Supplementary Fig. 2a–c, all the tested non-phosphorylatable SA and phosphomimetic SD mutants of STAT5A showed similar mitochondrial localization and interaction with PDC as STAT-WT, indicating that these phosphorylation sites are not necessary for the aforementioned process.

Taken together, these results indicate that mitochondria are the main cellular compartment where STAT5A and PDC interact.

### Mitochondrial STAT5A suppresses PDC activity and promotes the Warburg effect

**Fig. 3:**
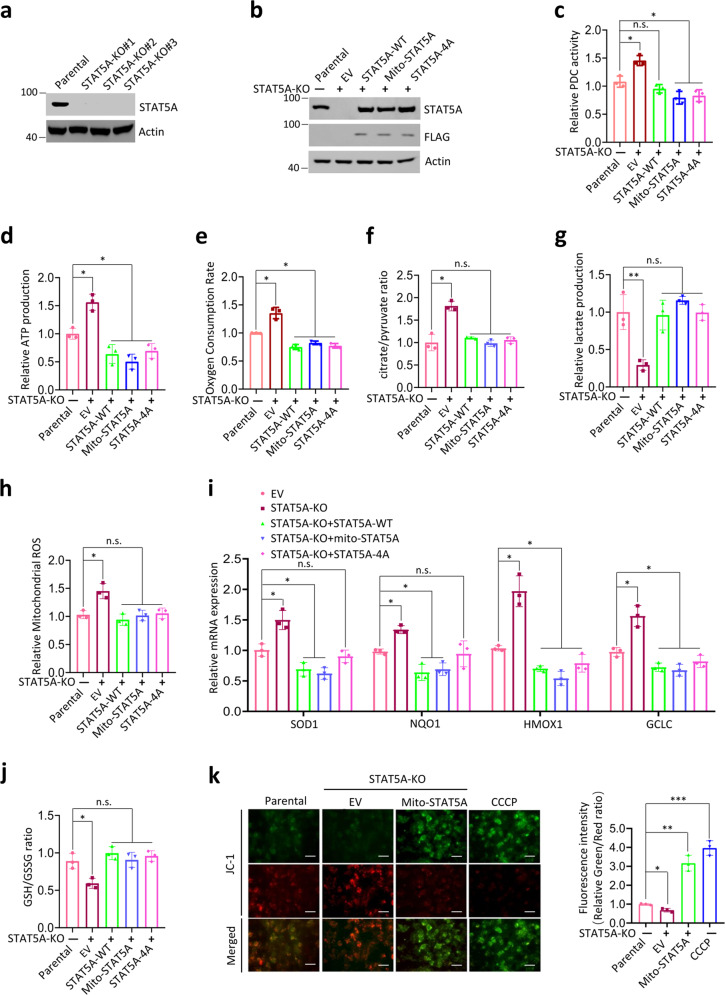
Mitochondrial STAT5A inhibits PDC activity and OXPHOS as well as promotes the Warburg effect in a transcription-independent manner. **a** Immunoblots of the indicated proteins in WCL from HeLa cells with STAT5A KO using CRISPR/Cas9 methods. Parental HeLa cells were used as the control. **b** The indicated expression vectors were stably transfected into STAT5A-KO HeLa cells. The proteins were immunoblotted with STAT5A antibody and antibody specific to the FLAG (for STAT5A). **c** STAT5A-KO HeLa cells were stably transfected with indicated expression vectors. The PDC activity levels were measured by in vitro PDC enzyme assays. Parental HeLa cells were used as the control. All data shown are mean values ± SD (error bar) from three independent experiments. **d** STAT5A-KO HeLa cells were stably transfected with indicated expression vectors. The Intracellular ATP levels were measured by ATP Assay Kit. Parental HeLa cells were used as the control. All data shown are mean values ± SD (error bar) from three independent experiments. **e** STAT5A-KO HeLa cells were stably transfected with indicated expression vectors. The oxygen-consumption rates were measured by Seahorse assays. Parental HeLa cells were used as the control. All data shown are mean values ± SD (error bar) from three independent experiments. **f** STAT5A-KO HeLa cells were stably transfected with indicated expression vectors. The citrate and pyruvate levels were measured by GC-MS. The citrate/pyruvate ratio was calculated. Parental HeLa cells were used as the control. All data shown are mean values ± SD (error bar) from three independent experiments. **g** STAT5A-KO HeLa cells were stably transfected with indicated expression vectors. The Intracellular lactate levels were measured by GC-MS. Parental HeLa cells were used as the control. All data shown are mean values ± SD (error bar) from three independent experiments. **h** STAT5A-KO HeLa cells were stably transfected with indicated expression vectors. The mitochondrial ROS levels were measured by mito-ROS indicator and analyzed by flow cytometry. Parental HeLa cells were used as the control. All data shown are mean values ± SD (error bar) from three independent experiments. **i** STAT5A-KO HeLa cells were stably transfected with indicated expression vectors. The mRNAs levels of oxidative stress-related genes were measured by RT-qPCR. Parental HeLa cells were used as the control. **j** STAT5A-KO HeLa cells were stably transfected with indicated expression vectors. The intracellular GSH and GSSG levels were measured by enzymatic assays. The GSH/GSSG ratio was calculated. Parental HeLa cells were used as the control. **k** STAT5A-KO HeLa cells were stably transfected with indicated expression vectors. The mitochondrial membrane potential levels were measured by JC-1 staining and the representative pictures are shown. Parental HeLa cells were used as the control. Red fluorescence represents a normal membrane potential and green fluorescence indicates mitochondrial membrane depolarization. CCCP treatment was used as a positive control for depolarized mitochondria. Scale bar = 20 μm. Fluorescence intensity was quantified by ImageJ. For statistical analysis, all data shown are mean values ± SD (error bar) from three independent experiments.

We then investigated whether the interaction between mitochondrial STAT5A and PDC would have any impact on PDC activity. First, STAT5A knockout (KO) HeLa cells were generated by CRISPR/Cas9-mediated genome editing methods. As shown in Fig. 3a, endogenous STAT5A expression was completely ablated in several single-cell clones. To exclude the possibility that the impact of STAT5A on PDC activity was mediated indirectly by nuclear STAT5A as a transcription factor, we generated three cell lines that stably overexpressing STAT5A-WT, mito-STAT5A, or STAT5A-4A mutant, in STAT5A-KO HeLa cells (Fig. 3b). STAT5A-4A mutant was constructed by mutating four key amino acids in the DNA-binding domain of STAT5A to Alanine (Fig. 2e). STAT5A-4A mutant was previously shown to not modulate transcription of its target genes because the mutations abolished the DNA-binding capacity of STAT5A [37]. Moreover, STAT5A-4A mutant is mostly cytoplasmic, and this distribution did not change following treatment with growth factors [38]. We found that PDC activity was increased in STAT5A-KO HeLa cells compared with parental cells (Fig. 3c). Reintroduction of STAT5A-WT into STAT5A-KO HeLa cells reduced PDC activity to levels similar to those in parental cells (Fig. 3c), thus excluding possible off-target effects of CRISPR/Cas9 knockout. Reintroduction of mito-STAT5A into STAT5A-KO HeLa cells also reversed the increase of PDC activity caused by STAT5A KO (Fig. 3c), indicating that mitochondrial STAT5A, but not nuclear STAT5A, suppressed the PDC activity. Reintroduction of STAT5A-4A into STAT5A-KO HeLa cells reversed the upregulation of PDC activity caused by STAT5A KO (Fig. 3c), indicating that STAT5A suppressed PDC activity in a transcription activity-independent manner. We also found that STAT5A KO led to increased ATP production (Fig. 3d), oxygen-consumption rate (Fig. 3e), citrate/pyruvate ratio (Fig. 3f), and decreased lactate production (Fig. 3g), indicating decreased glycolysis and increased mitochondrial oxidative metabolism in STAT5A-KO HeLa cells. PDC is critical for the conversion of pyruvate to acetyl-CoA and the promotion of OXPHOS and the production of ROS. We found that STAT5A KO led to increased mitochondrial ROS production (Fig. 3h), induction of antioxidant genes (Fig. 3i), and a decrease in the reduced/oxidized glutathione ratio (Fig. 3j). Moreover, these metabolic changes in STAT5A-KO cells were largely reversed by reintroduction of STAT5A-4A or mito-STAT5A (Fig. 3c–j). We also found that STAT5A KO led to increased mitochondrial membrane potential which were reversed by reintroduction of mito-STAT5A (Fig. 3k), Taken together, these results indicate that mitochondrial STAT5A inhibits PDC activity and promotes the Warburg effect in a transcription-independent manner.

### Mitochondrial STAT5A destabilizes PDC integrity

**Fig. 4:**
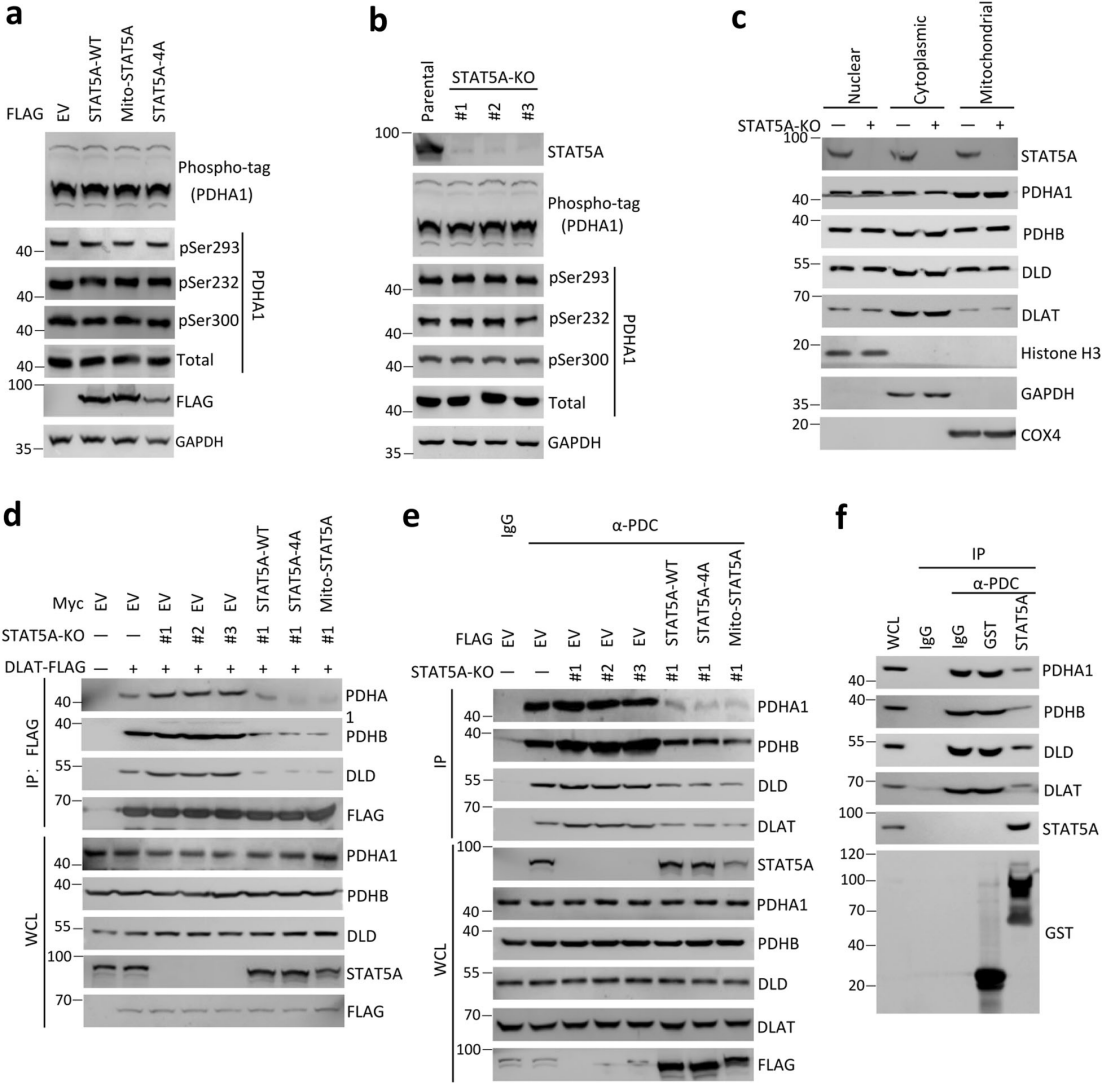
Mitochondrial STAT5A suppresses PDC formation. **a** HeLa cells were stably transfected with indicated expression vectors. The WCL were immunoblotted with indicated antibodies. The phosphorylated level of PDHA1 (p-PDHA1) was detected by phos-tag SDS-PAGE using an anti-PDHA1 antibody. **b** Immunoblots of the indicated proteins in WCL from parental or STAT5A-KO HeLa cells using CRISPR/Cas9 methods. The phosphorylated level of PDHA1 (p-PDHA1) was detected by The WCL were immunoblotted with indicated antibodies. Phos-tag SDS-PAGE was performed using an anti-PDHA1 antibody. Parental HeLa cells were used as the control. **c** The nuclear, cytoplasmic, and mitochondrial fractions from parental and STAT5A-KO HeLa cells. The fractionated proteins were immunoblotted with indicated antibodies. **d** STAT5A-KO HeLa cells were stably transfected with indicated expression vectors. Then, the cells were transiently transfected with expression vectors encoding DLAT-FLAG. WCL were immunoprecipitated with anti-FLAG antibody, and the immunoprecipitated proteins were immunoblotted with indicated antibodies. **e** STAT5A-KO HeLa cells were transfected with indicated expression vectors. Cell lysates were immunoprecipitated with anti-PDC immunocapture antibody, and the proteins were immunoblotted with indicated antibodies. IgG was used as a negative control for Immunoprecipitation. **f** The intact PDC was immunoprecipitated from HeLa cells by anti-PDC immunocapture antibody and then incubated with the indicated recombinant proteins (GST or GST-STAT5A, 2 μg) for 12 h. Bound materials and recombinant proteins were detected by Immunoblot.

We then investigated the underlying molecular mechanisms by which mitochondrial STAT5A inhibits PDC activity. A well-known regulatory model of PDC function involves inhibitory serine phosphorylation of PDHA1 by PDH kinases (PDK1**–**4), whereas dephosphorylation of PDHA by PDH phosphatases (PDP1**–**2) activates PDC (refs. [39, 40]). Our TAP-MS results showed that PDK2 and PDK3 were present in the STAT5A complexes (Fig. 2a), which led us to investigate whether STAT5A affects PDHA1 phosphorylation. However, ectopically overexpressed STAT5A-WT, mito-STAT5A, or STAT5A-4A mutant had no impact on PDHA1 phosphorylation, as detected by Immunoblot analyses with three phospho-specific PDHA1 antibodies (pSer232, pSer293, and pSer300) or using a phospho-tag gel (Fig. 4a). The level of PDHA1 phosphorylation was comparable between parental and STAT5A-KO HeLa cells (Fig. 4b). STAT5A KO did not alter the relative distributions of PDC subunits in three subcellular fractions (Fig. 4c). Therefore, we sought to investigate an alternative possibility that STAT5A affects PDC integrity by reducing protein–protein interaction between the subunits. We detected endogenous PDHA1, PDHB, and DLD immunoprecipitated by FLAG-DLAT. As shown in Fig. 4d, STAT5A KO led to a marked increase in PDC subunits immunoprecipitated by DLAT. However, this effect was reversed by reintroduction of STAT5A-WT, mito-STAT5A, or STAT5A-4A mutant into STAT5A-KO HeLa cells (Fig. 4d). We also used PDC immunocapture beads to immunoprecipitate endogenous PDC. An increase in the immunoprecipitated PDC was observed in STAT5A-KO HeLa cells compared with parental HeLa cells, but this effect can be reversed by reintroduction of STAT5A-WT, mito-STAT5A, or STAT5A-4A into STAT5A-KO HeLa cells (Fig. 4e). Moreover, when the immunoprecipitated PDC was incubated with recombinant STAT5A, the amounts of intact PDC immunoprecipitated by PDC immunocapture beads were markedly decreased (Fig. 4f). Taken together, these data indicate that mitochondrial STAT5A destabilizes PDC integrity.

### Mitochondrial STAT5A is involved in hypoxia-mediated PDC inactivation and metabolic remodeling

**Fig. 5:**
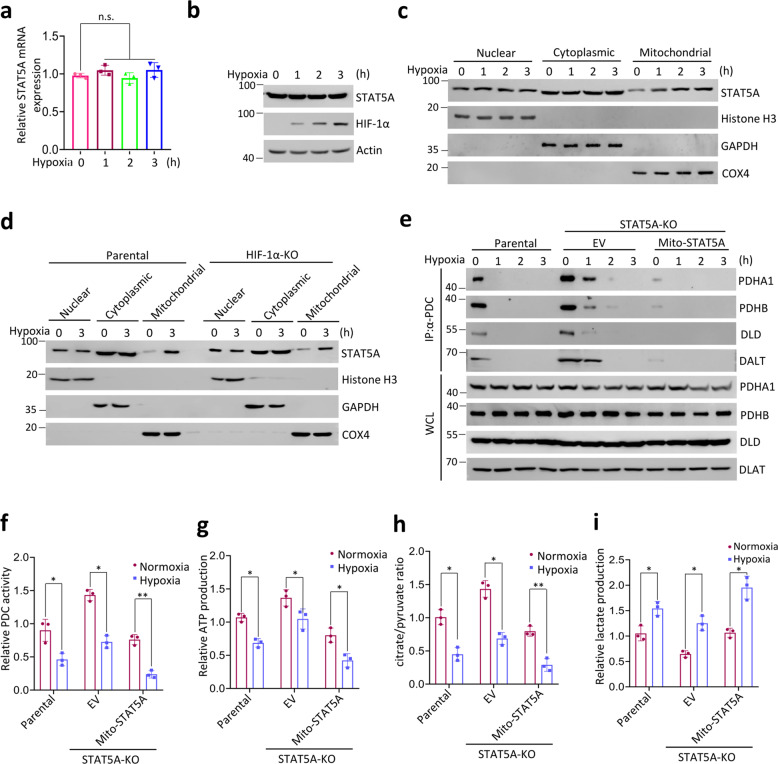
STAT5A is involved in hypoxia-induced PDC inactivation. **a** RT-qPCR assessment of STAT5A mRNA expression in HeLa cells under hypoxic (1% O_2_) condition for the indicated times. **b** Immunoblots of the indicated proteins in WCL from HeLa cells under hypoxic (1% O_2_) condition for the indicated times. **c** HeLa cells were under hypoxic (1% O_2_) conditions for the indicated times. The nuclear, cytoplasmic, and mitochondrial fractions were prepared for Immunoblot. **d** Parental and HIF1α-KO HeLa cells were under hypoxic (1% O_2_) conditions for the indicated times. The nuclear, cytoplasmic, and mitochondrial fractions were prepared for Immunoblot. **e** STAT5A-KO HeLa cells were stably transfected with expression vectors encoding EV or FLAG-Mito-STAT5A under hypoxic (1% O_2_) conditions for the indicated times. WCL were immunoprecipitated with an anti-PDC immunocapture antibody, and the proteins were immunoblotted with indicated antibodies. Parental HeLa cells were used as the control. **f** STAT5A-KO HeLa cells were transfected with expression vectors encoding EV or FLAG-Mito-STAT5A under normoxic or hypoxic (1% O_2_) conditions for 3 h. The PDC activity levels were measured by in vitro PDC enzyme assays. Parental HeLa cells were used as the control. **g** STAT5A-KO HeLa cells were transfected with expression vectors encoding EV or FLAG-Mito-STAT5A under normoxic or hypoxic (1% O_2_) conditions for 3 h. The Intracellular ATP levels were measured by ATP Assay Kit. Parental HeLa cells were used as the control. **h** STAT5A-KO HeLa cells were transfected with expression vectors encoding EV or FLAG-Mito-STAT5A under normoxic or hypoxic (1% O_2_) conditions for 3 h. The citrate and pyruvate levels were measured by GC-MS. The citrate/pyruvate ratio was calculated. Parental HeLa cells were used as the control. **i** STAT5A-KO HeLa cells were transfected with expression vectors encoding EV or FLAG-Mito-STAT5A under normoxic or hypoxic (1% O_2_) conditions for 3 h. The Intracellular lactate levels were measured by GC-MS. Parental HeLa cells were used as the control. For statistical analysis, all data shown are mean values ± SD (error bar) from three independent experiments.

Mitochondrial PDC activity is dynamically regulated in response to various stresses [9]. In hypoxic conditions, cancer cell metabolism undergoes a shift from OXPHOS to glycolysis partially by inhibiting PDC from using pyruvate to fuel the TCA cycle [3, 4]. We investigated whether mitochondrial STAT5A is involved in hypoxia-induced PDC inactivation. Although the mRNA and protein levels of STAT5A remained unaltered (Fig. 5a, b), hypoxia treatment led to an increase in the amount of mitochondrial STAT5A in a time-dependent manner (Fig. 5c). Hypoxia-enhanced mitochondrial translocation was also observed in HIF1-α KO HeLa cells at a similar level to that in parental HeLa cells, indicating that this process acts in an HIF1-α-independent manner (Fig. 5d). By contrast, EGF treatment led to a marked increase in the amounts of nuclear STAT5A, but had no impact on the amounts of mitochondrial STAT5A (Supplementary Fig. 3). Hypoxia treatment led to a rapid disruption of PDC integrity as demonstrated by the reduction of immunoprecipitated PDC subunits. However, this effect was attenuated in STAT5A-KO HeLa cells, but was potentiated in STAT5A-KO HeLa cells reconstituted with mito-STAT5A (Fig. 5e). Moreover, hypoxia-induced metabolic remodeling of PDC activity (Fig. 5f), ATP production (Fig. 5g), citrate/pyruvate ratio (Fig. 5h), and lactate production (Fig. 5i) were blunted in STAT5-KO HeLa cells compared with parental HeLa cells, all of which were largely reversed by reintroduction of mito-STAT5A (Fig. 5f–i). Taken together, these data indicate that mitochondrial STAT5A is involved in hypoxia-mediated PDC inactivation and metabolic remodeling.

### Mitochondrial STAT5A supports in vitro cell proliferation under hypoxia and in vivo tumor growth

**Fig. 6:**
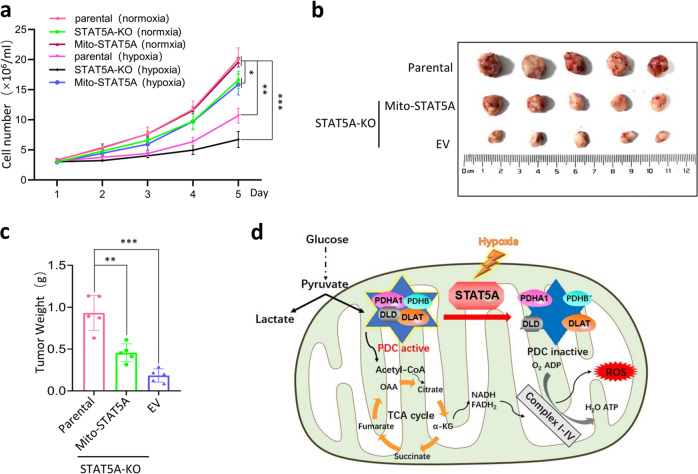
Mitochondrial STAT5A supports cell proliferation in vitro under hypoxia and tumor growth in vivo. **a** STAT5A-KO HeLa cells were stably transfected with indicated expression vectors. The cells were under normoxic or hypoxic (1% O_2_) conditions for indicated days. Parental HeLa cells were used as the control. The cell proliferation analysis was measured by Cell Counting Kit-8 (CCK-8). **b**, **c** Parental or STAT5A-KO HeLa cells stably overexpressing EV or Mito-STAT5A were implanted subcutaneously in nude mice. After 3 weeks, the tumors dissected from nude mice were photographed for comparison (**b**). The weight of implanted tumors (**c**). Five mice were used in each group (*n* = 5). **d** A proposed model shows that mitochondrial STAT5A acts as a regulator of PDC activity. For statistical analysis, all data shown are mean values ± SD (error bar) from three independent experiments.

Lastly, we determined whether mitochondrial STAT5A has any impact on cancer cell growth in vitro and in vivo. We compared the cell growth rates of parental HeLa cells, STAT5A-KO HeLa cells, and STAT5A-KO HeLa cells reconstituted with mito-STAT5A under normoxia and hypoxia. Under normoxic conditions, STAT5A KO led to a significant reduction of cell growth compared with the growth of parental HeLa cells, consistent with the notion that STAT5A is an oncoprotein. However, no significant difference in cell growth was observed between STAT5A-KO HeLa cells and STAT5A-KO HeLa cells reconstituted with mito-STAT5A under normoxic conditions. By contrast, when these cells were switched to hypoxic conditions, STAT5A-KO HeLa cells reconstituted with mito-STAT5A were more resistant to hypoxia-mediated cell growth suppression compared with STAT5A-KO HeLa cells (Fig. 6a). In vivo xenograft tumor assays showed STAT5A KO led to a profound reduction of in vivo tumor growth, which was partially compensated by mito-STAT5A reintroduction (Fig. 6b, c). Taken together, these data indicate that mitochondrial STAT5A positively regulates in vitro cell proliferation under hypoxia conditions and in vivo tumor growth.

## Discussion

In this study, we demonstrate that mitochondria are the major cellular compartment in which the STAT5A–PDC interaction occurs. Mitochondrial STAT5A disrupts PDC integrity, thereby inhibiting PDC activity, and remodeling cellular glycolysis and OXPHOS. Under hypoxia, the translocation of STAT5A to mitochondria was increased to further strengthen the Warburg effect and support in vitro cell growth and in vivo tumor growth. Our study indicates a pro-oncogenic role of STAT5A in energy metabolism, which is different from its classical function as a transcription factor (Fig. 6d). A moderate interaction between STAT and PDC was detected, it is not surprising since only a small pool of STAT5A is localized in the mitochondria. In addition to mitochondria, we detected a minimal STAT5A–PDC interaction occurring in the nuclei. The functional relevance of this interaction was not determined in this study, but a recent report suggested that PDC may modulate the ability STAT5A to regulate gene expression by controlling histone or STAT5A acetylation [41].

In this study, we found that hypoxia treatment enhanced STAT5A mitochondrial translocation. A similar trend was observed in HIF1α-KO HeLa cells, indicating that this process acts in a HIF1α-independent manner. STAT5A does not have a canonical mitochondrial targeting sequence, therefore, the mechanism underlying mitochondrial translocation of STAT5A remains obscure. A recent study suggested that GRIM19, a complex I subunit located in the inner mitochondrial membrane, acts as a chaperone to recruit STAT3 into the mitochondria [42]. It is unclear whether a similar mechanism is at play for STAT5A. A previous report showed that STAT5 was only detected in the mitochondrial fractions of CTLL-20 cells after Interleukin-2 stimulation. Tyrosine-phosphorylated STAT5 was also significantly more abundant in the mitochondrial fraction [43]. However, we did not observe any change in the amounts of mitochondrial STAT5A between EGF-treated and non-treated HeLa cells, although strong tyrosine phosphorylation of STAT5A was observed upon EGF treatment (Supplementary Fig. 2). The discrepancy between the two studies is unexplained but may be caused by the different cell systems and experimental conditions used. Moreover, the detailed molecular mechanisms through which STST5A disrupt PDC integrity is still unknown now, but our current results demonstrate this process is independent of PDHA1 phosphorylation. A previous study showed that prolyl-hydroxylase 3 (PHD3) interacts with PDC, and maintains PDC integrity independent of its enzymatic activity [44]. Thus, modulation of the modular PDC architecture by various regulators is common, in addition to regulation by phosphorylation. Further investigation is required to elucidate the exact pathway by which hypoxia-induced oncogenic transformation and metabolism conversion occur through the mitochondrial STAT5A. We suggest further studies are warranted to explore and develop inhibitors targeting STAT5A for tumor suppression.

## Materials and methods

### Cell culture and transfections

293T, HeLa, and MIHA (an immortalized human hepatocyte cell line) cells were maintained in DMEM with 10%(v/v) FBS. The cell line authenticity and mycoplasma infection are routinely checked by DNA fingerprinting and PCR. Primary mouse hepatocytes were obtained from mice with the collagenase perfusion method. The cells were cultured in M199 media with 10% FBS n on collagen-coated plates. The culture media was replaced with fresh media 3 h after attachment. All cells were cultured at 37 °C in an atmosphere of 5% CO_2_. For transient transfection, cells were transfected with PEI (Polyethyleneimine) or Lipofectamine 2000 (Thermo) following the manufacturer’s instructions. For lentiviral transfection, the pCDH plasmids carrying target constructs, pGAG and pVSVG plasmids, were co-transfected into 293T cells. A virus-containing supernatant was harvested 48 h after transfection to infect HeLa cells with 10 μg/ml polybrene.

### CRISPR–Cas9-mediated gene knockout stable cell generation

pX459 plasmid was used to clone guide oligos targeting STAT5A or HIF1A gene. Plasmids containing the target guide sequence were cloned following the protocol of Zhang lab (https://www.addgene.org/crispr/zhang/). HeLa cells were plated and transfected with pX459 constructs overnight. Twenty-four hours after transfection, 1 μg/ml puromycin was used to screen cells for 3 days. Living cells were seeded in a 96-well plate by limited dilution to isolate monoclonal cell lines. The knockout cell clones were screened by Immunoblot and validated by Sanger sequencing. Sequences of gene-specific sgRNAs are listed in Supplementary Table 1.

### Real-time reverse transcription PCR (RT-qPCR)

Total RNA was isolated from cells using the TRIzol reagent (Tiangen), and cDNA was reversed-transcribed using the Superscript kit (Vazyme) following the manufacturer’s instructions. PCR amplification was performed using the ChamQ SYBR qPCR Master Mix (Vazyme). All quantitation was normalized to the levels of control GAPDH. The sequences of Primers for RT-qPCR are listed in Supplementary Table 1.

### Co-immunoprecipitation

To immunoprecipitate ectopically expressed FLAG-tagged proteins, transfected cells were lysed 24 h after transfection in 0.1% NP40 buffer. The whole-cell lysates were immunoprecipitated with monoclonal anti-FLAG antibody-conjugated agarose beads (Sigma-Aldrich) at 4 °C overnight. After three washes with lysis buffer followed by two washes with 0.1% NP40 buffer, the bound proteins were eluted using FLAG peptide (Sigma-Aldrich) prepared in 0.1% NP40 for 3 h at 4 °C. The eluted protein samples were resolved by SDS-PAGE. To immunoprecipitate endogenous proteins, cells were lysed with 1 × cell lysis buffer (Cell Signaling Technology), and the lysates were centrifuged. The supernatants were precleared with Protein A/G beads (Sigma-Aldrich) and incubated with the indicated antibody and Protein A/G beads at 4 °C overnight. Beads were washed five times with lysis buffer, resuspended in sample buffer, and analyzed by SDS-PAGE.

### Immunoblot

Cell lysates or immunoprecipitates were subjected to SDS-PAGE, and proteins were transferred to nitrocellulose membranes (GE Healthcare Sciences). Membranes were blocked in Tris-buffered saline (TBS, pH 7.4) containing 5% nonfat milk and 0.1% Tween-20, washed twice in TBS containing 0.1% Tween-20, and incubated with primary antibody overnight at 4 °C followed by the secondary antibody for 1 h at room temperature. Proteins of interest were visualized using the Enhanced Chemiluminescence (ECL) system (Santa Cruz Biotechnology). Densitometry analysis of protein bands was performed on Gel-Pro Analyzer software. The resources and other information on antibodies are listed in Supplementary Table 1.

### Isolation of nucleus, cytoplasm, and mitochondria

HeLa cells were prepared for nuclear, cytoplasmic, and mitochondrial extraction by density-gradient centrifugation. Briefly, HeLa cells were washed three times with PBS. Then the cells are suspended by using hypotonic solution (140 mM KCl, 10 mM EDTA, 5 mM MgCl_2_, 20 mM HEPES (pH 7.4), and the protease inhibitor). Then 5 × 10^6^ HeLa cells were ground with a glass homogenizer in an ice bath for 25 strokes. Nuclear, cytoplasmic, and mitochondrial fractions were separated through differential centrifugation (800×*g*, 10 min, 4 °C and 12,000×*g*, 35 min, 4 °C). The supernatant (cytoplasmic fraction) and pellet (mitochondrial fraction) were collected, and the pellet was further washed with wash buffer (800 mM KCl, 10 mM EDTA, 5 mM MgCl_2_, and 20 mM HEPES (pH 7.4), and the protease inhibitor) for three times and yield the final mitochondrial fraction. To confirm that pure extracts were obtained, the mitochondrial, nuclear, and cytoplasmic fractions were separated by SDS-PAGE, and the presence of mitochondrial VDAC1, BCL2, nuclear Histone H3, and cytoplasmic GAPDH was detected by Immunoblot. The band intensity of the indicated proteins was quantified by ImageJ software. According to band intensity quantification and fraction amounts, the distribution of the indicated proteins in the three fractions was calculated.

The same amount of FLAG-STAT5A in three separated fractions were subjected to co-IP assays and detected by immunoblot. The method is as follows: immunoblot was used to quantify the STAT5A proteins in the three fractions. The amounts of STAT5A proteins in the mitochondrial fraction were taken as a reference, and the amounts of STAT5A protein in the nuclear fraction or cytoplasmic fraction were adjusted to be consistent with that in the mitochondrial fraction.

### Mitochondrial protein-localization assays

Mitochondria were purified by the methods mentioned above. The control group was not treated. The second group was treated with proteinase K (3 μM). The third group was treated with proteinase K (3 μM) and 0.1–0.5% Triton-X-100 solution. Three groups of samples were placed in a 37 °C water bath for 30 min. The samples were prepared and then detected by immunoblot.

### Oxygen-consumption rate assays

Oxygen-consumption rate (OCR) was measured under basal conditions in the presence of the mitochondrial inhibitors oligomycin (0.5 μM, Calbiochem). OCR was calculated by the oligomycin-induced changes in comparison to basal rates. The total protein of each well was determined by Bradford assays and used as the reference to normalize the OCR. OCR was measured using a Seahorse XF96 extracellular flux analyzer (Seahorse Bioscience).

### ATP production assays

Cellular ATP contents were measured according to the instruction manual for the reagent (Beyotime). Cells were lysed with 100 μl lysis buffer from the assay kit. The supernatant was collected after centrifugation at 12,000×*g*, 4 °C for 5 min and used to detect ATP levels; 50 μl of ATP-detection working solution was added to each tube before detection for 5 min to completely consume the original ATP. The firefly luciferase activity of each sample, expressed in RLU (relative light unit), was measured for at least 2 s with a GLOMA luminometer (Promega) with 20 μl of cell lysates added to each tube.

### PDC activity assays

Cells were washed and harvested in PBS. Proteins were extracted by ultrasonic breaking and centrifugation. Pyruvate dehydrogenase enzyme activity assays were performed using the PDC Activity Microplate Assay Kit (Abcam) following the manufacturer’s instructions. Briefly, samples containing PDH complex were prepared, loaded onto 96-well plates coated with anti-PDH monoclonal antibody, and incubated for 3 h at RT. A volume of 200 μL of assay solution was added to each well, optical density (OD 450 nm) was measured in kinetic mode at RT for 30 min, and finally the relative activity of the PDH complex was calculated. Rate (mOD/min) = absorbance 2–absorbance 1/time (minutes). The relative PDC activity is calculated by normalizing the rate of treated groups to the rate of control groups.

### Mitochondrial membrane potential assays

Mitochondrial membrane potential was determined by a mitochondrial membrane potential assay kit with JC-1(Beyotime). The mitochondrial membrane potential can be determined by comparing the intensity of two kinds of light emissions. HeLa cells seeded in 12-well cell culture plates were treated as described and then stained with JC-1 according to the manufacturer’s instructions. Briefly, 6 × 10^5^ HeLa cells were harvested, fixed with 0.5 ml of cell culture media and 0.5 ml of JC-1 staining working solution, and incubated in a cell culture cabinet for 20 min after gentle shaking. Cells were collected through centrifugation at 600×*g*, 4 °C for 3 min and then washed three times with 1xJC-1 buffer solution. The cell fluorescence intensity was measured with a microplate reader in 96-well plates after cells were resuspended in 100 μl of 1xJC-1 buffer solution.

### Gas chromatography–mass spectrometry analysis (GC-MS)

HeLa cells were washed with ice-cold PBS and harvested with −80 °C cold 80% methanol and stored at −80 °C for 1 h. The supernatants were collected by centrifugation and lyophilized in glass bottles overnight. The samples were oximated by 20 mg/ml methoxyamine hydrochloride in pyridine at 70 °C for 90 min and derived in 20% N-methyl-N-(tert-butyldimethylsilyl) trifluoroacetamide dissolved in pyridine at 30 °C for 30 min. The samples were filtrated with 2 μl filter before subjected to GC-MS analysis (Agilent Technologies 7890B). The signals of lactate, pyruvate, and citrate were determined through their specific ion peaks. The amounts of the corresponding molecules were calculated by Integrating the signal value and retention time value. The values are normalized to the control groups.

### Cell proliferation assays

Cell proliferation rate was determined using Cell Counting Kit-8 (CCK-8) according to the manufacturer’s protocol (Beyotime). Briefly, cells were seeded onto 96-well plates at a density of 1000 cells per well. During a 1–5-day culture period, 10 μl of the CCK-8 solution was added to cell culture and incubated for 2 h. The resulting color was assayed at OD 450 nm using a microplate absorbance reader. Each assay was carried out in triplicate.

### Xenograft tumor assays

All experimental protocols were approved in advance by the Ethics Review Committee for Animal Experimentation of Fudan University. 4–6-week-old BALB/c nu/nu mice obtained from SLAC Laboratory Animal Co., Ltd. were bred and maintained in our institutional pathogen-free mouse facilities. In total, 5 × 10^6^ parental HeLa cells or STAT5A-KO HeLa cells stably overexpressing EV or mito-STAT5A were suspended in 100 μl of PBS buffer and injected into the flanks of male nude mice (four mice for each group). At the end of 3 weeks, mice were killed and in vivo solid tumors were dissected and weighed.

### Statistics analysis

The statistical calculations were performed using Graph-Pad Prism software. All data are shown as mean values ± SD for experiments performed with at least three replicates. The difference between the two groups was analyzed using paired Student’s *t* test unless otherwise specified. *P < 0.05; **P < 0.01; ***P < 0.001.

## Supplementary information

Supplementary Figures and Table
