# Supplementary material for: Mitochondrial STAT5A promotes metabolic remodeling and the Warburg effect by inactivating the pyruvate dehydrogenase complex
Source: Cell Death Dis. 2021 Jun 19;12(7):634. doi: 10.1038/s41419-021-03908-0 (PMC8214628; doi:10.1038/s41419-021-03908-0)
Supplement: Supplementary file 1 — Supplementary Figures and Table [file 41419_2021_3908_MOESM1_ESM.docx]

Supplementary Materials for

**Mitochondrial STAT5A promotes metabolic remodeling and the Warburg effect by inactivating the pyruvate dehydrogenase complex**

Liang Zhang, Jianong Zhang, Yan Liu, Pingzhao Zhang, Ji Nie, Rui Zhao, Qin Shi, Huiru Sun, Dongyue Jiao, Yingji Chen, Xiaying Zhao, Yan Huang, Yao Li, Jian-Yuan Zhao, Wei Xu, Shi-Min Zhao and Chenji Wang


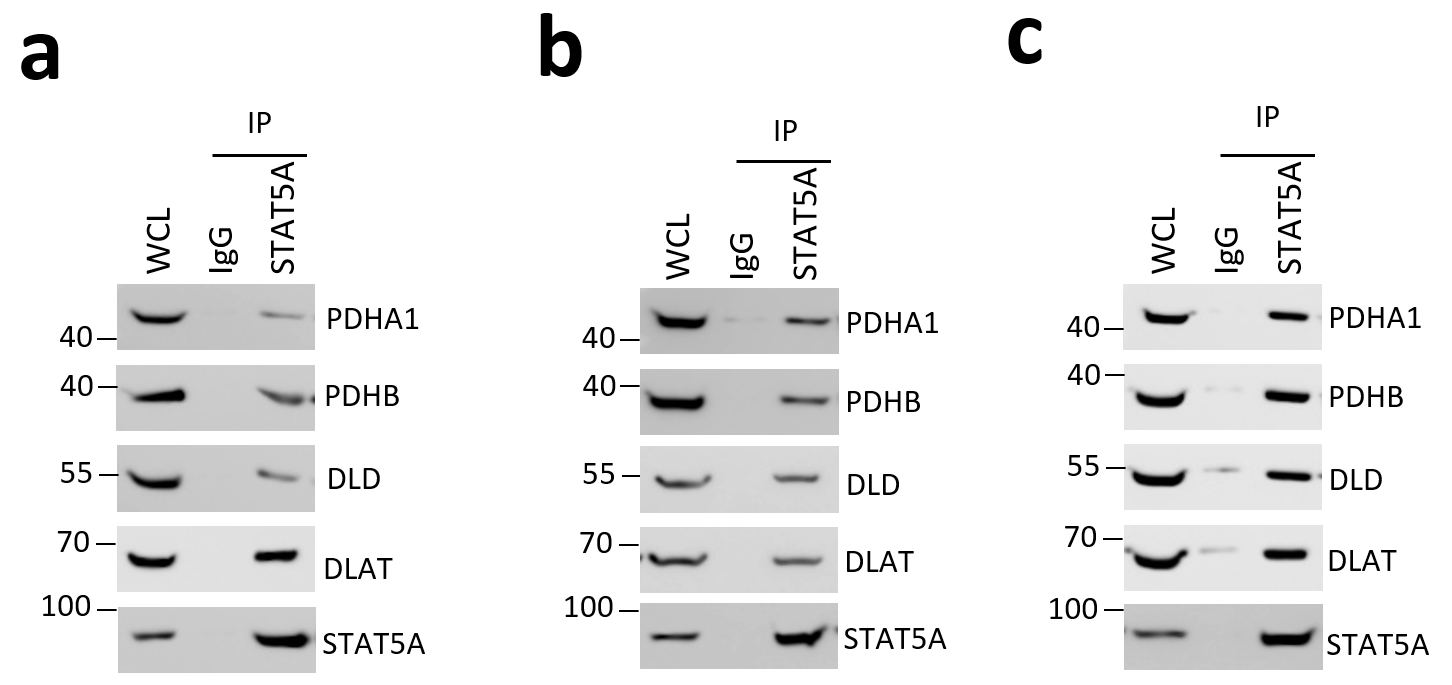


**Supplementary Figure 1. Endogenous STAT5A interact with PDC in cancer cells.** Co-immunoprecipitation of STAT5A with PDC from HeLa (**a**) MIHA (**b**) or primary mouse Hepatocytes (**c**) cells. Whole-cell extracts (lane 1) or immunoprecipitates generated with the STAT5A antibody (lane 3) or a control IgG (lane 2) were immunoblotted with indicated antibodies. The total protein used for immunoprecipitation was 10-12 mg. The protein used for blotting was 45-60 μg per Lane.


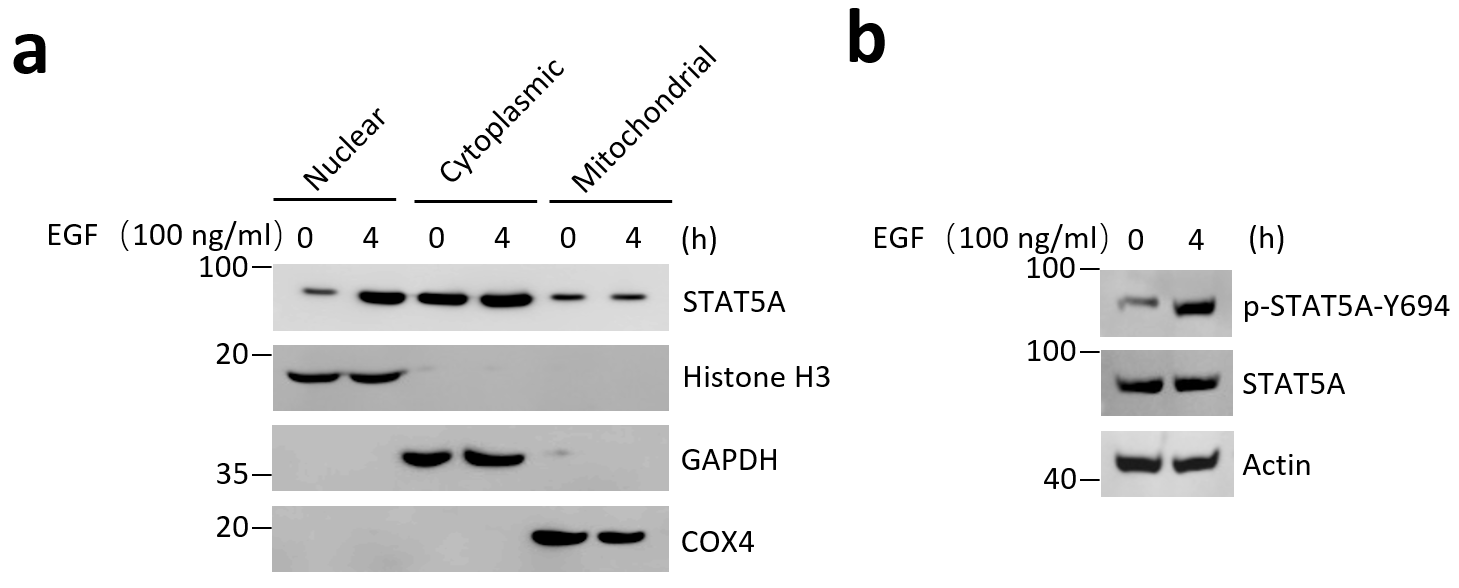


**Supplementary Figure 2. EGF treatment had no impact on the mitochondrial translocation of STAT5A.** (**a**) HeLa cells were under EGF treatment for the indicated times. Cell lysates of the nuclear, cytoplasmic, and mitochondrial fractions were prepared for western blotting analysis of STAT5A (upper panel). The total protein used for isolation of subcellular fraction was 4-5 mg. (**b**) Western blots of the indicated proteins in WCL from HeLa cells under EGF treatment for the indicated times. The proteins were immunoblotted with antibody specific to the p-STAT5A-Y694 (upper panel) and STAT5A epitope.


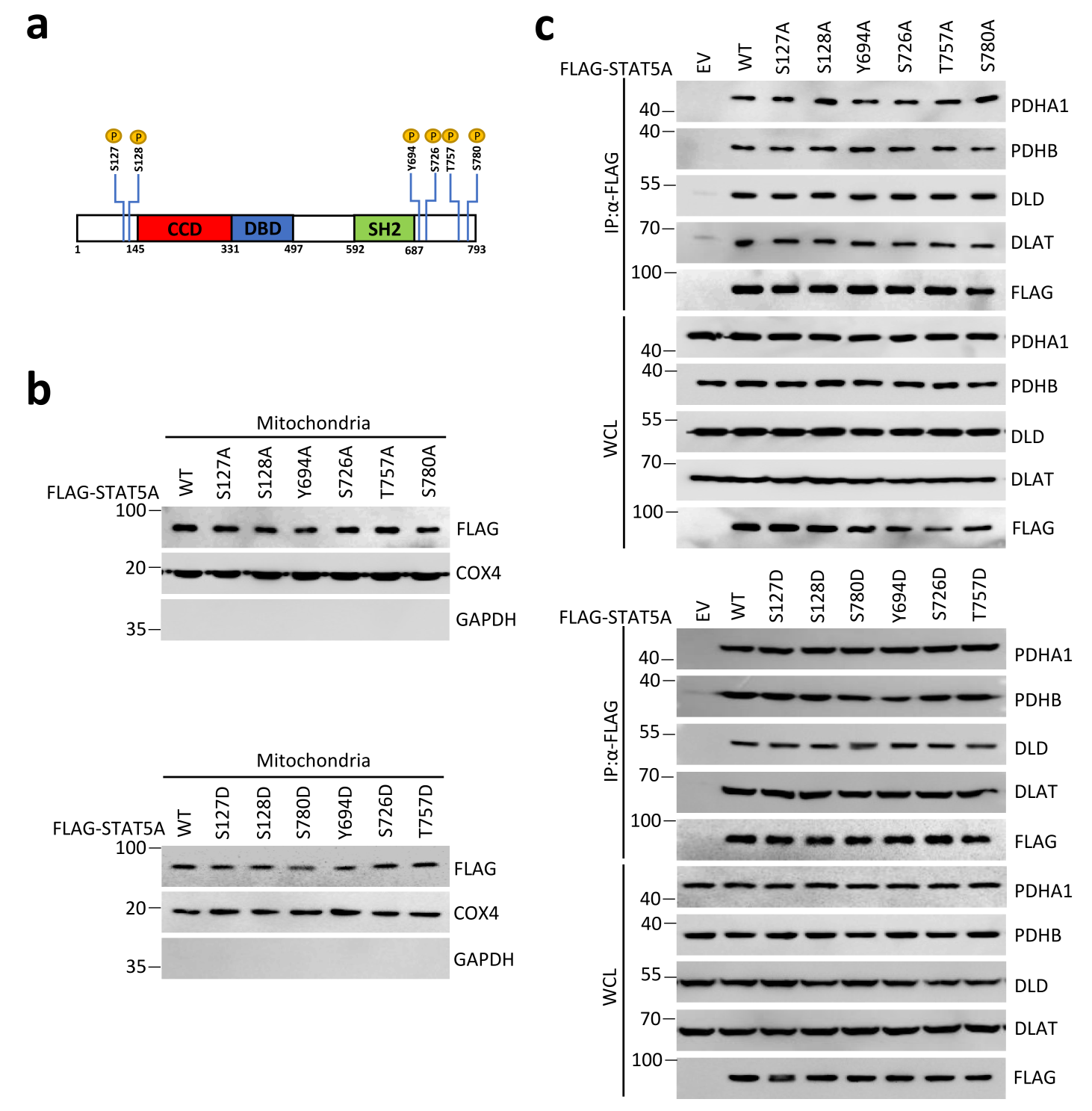


**Supplementary Figure 3. The nonphosphorylatable and phosphomimetic mutants of STAT5A show comparable mitochondrial localization and interaction with PDC as STAT5A-WT.** (**a**) Distribution of the phosphorylation site of STAT5A. (**b**) Western blots of the indicated proteins in mitochondrial fractions from HeLa cells transfected with indicated plasmids. The mitochondrial fraction from HeLa cells transfected with expression vectors encoding indicated proteins. The proteins were immunoblotted with antibody specific to the FLAG (for STAT5A; upper panel) epitope. The total protein used for isolation of mitochondria was 4-5 mg. (**c**) HeLa cells were transfected with expression vectors encoding indicated proteins. Lysates were immunoprecipitated with anti-FLAG antibody, and the proteins were immunoblotted with indicated antibodies. The total protein used for immunoprecipitation was 4-5 mg. The protein used for blotting per Lane was 20-30 μg.

**Supplementary Table 1**

| **Antibodies** | | | |
| --- | --- | --- | --- |
| REAGENT or RESOURCE | SOURCE | IDENTIFIER | Application/Dilutions |
| STAT5A | Cell Signaling Technology | Cat# 94205S | IB：1:1000  IP：1:50 |
| PDHA1 | Proteintech | Cat# 66119-1-Ig | IB：1:1000  IP：1:50 |
| PDHB | Abcam | Cat# ab155953 | IB：1:1000 |
| DLD | Abcam | Cat# ab133551 | IB：1:1000 |
| DLAT | Abcam | Cat# ab172617 | IB：1:1000 |
| PDH-E1α(pSer232) | Millipore | Cat# AP1063 | IB：1:1000 |
| PDH-E1α(pSer293) | Millipore | Cat# ABS204 | IB：1:1000 |
| PDH-E1α(pSer300) | Millipore | Cat# ABS194 | IB：1:1000 |
| HIF1-α | Cell Signaling Technology | Cat# 36169 | IB：1:1000 |
| Actin | Yeasen | Cat#30101ES10 | IB：1:5000 |
| GAPDH | Yeasen | Cat#30201ES20 | IB：1:5000 |
| Histone H3 | Cell Signaling Technology | Cat#9717 | IB：1:10000 |
| COX4 | Cell Signaling Technology | Cat#4850 | IB：1:1000 |
| VDAC1 | Abcam | Cat# ab14734 | IB：1:1000 |
| Bcl-2 | Cell Signaling Technology | Cat#4223S | IB：1:1000 |
| STAT5A-pY694 | Cell Signaling Technology | Cat#4322T | IB：1:1000 |
| FLAG | Abmart | Cat# M20008 | IB：1:5000 |
| Myc | Abmart | Cat# M20003 | IB：1:5000 |

| **Chemicals** | | |
| --- | --- | --- |
| REAGENT or RESOURCE | SOURCE | IDENTIFIER |
| DMEM | Gibco | Cat#11960044 |
| Fetal Bovine Serum | Gibco | Cat# 10099 |
| Penicillin-Streptomycin | Invitrogen | Cat#15070063 |
| methoxyamine hydrochloride | Sigma | Cat#226904 |
| N-methyl-N-(tert-butyldimethylsilyl) trifluoroacetamide | Sigma | Cat#394882 |
| protein-A Sepharose bead | Merck Millipore | Cat#16-156 |
| proteinase K | Tiangen | Cat#RT403 |
| [ChamQ SYBR qPCR Master Mix](http://www.vazymebiotech.com/products_detail/productId=99.html) | Vazyme Biotech Co., Ltd | Cat#Q311 |
| [Phanta Max Super-Fidelity DNA Polymerase](http://www.vazymebiotech.com/products_detail/productId=119.html) | Vazyme Biotech Co., Ltd | Cat#P505 |

| **Assay Kit** | | |
| --- | --- | --- |
| REAGENT or RESOURCE | SOURCE | IDENTIFIER |
| Pyruvate dehydrogenase (PDH) Enzyme Activity Microplate Assay Kit | Abcam | Cat#ab109902 |
| Mitochondrial membrane potential assay kit with JC-1 | Beyotime Technology | Cat#C2006 |
| ATP Assay Kit | Beyotime Technology | Cat#S0026 |
| BCA Protein Assay Kit | Beyotime Technology | Cat#P0012S |
| PDH immunocapture kit | Abcam | Cat#ab109802 |
| Cell Counting Kit-8 | Beyotime Technology | Cat#C0037 |
| GSH and GSSG Assay Kit | Beyotime Technology | Cat#S0053 |
| [ClonExpress II One Step Cloning Kit](http://www.vazymebiotech.com/products_detail/productId=89.html) | Vazyme Biotech Co., Ltd | Cat#C112 |
| [HiScript III 1st Strand cDNA Synthesis Kit (+gDNA wiper)](http://www.vazymebiotech.com/products_detail/productId=216.html) | Vazyme Biotech Co., Ltd | Cat#R312 |

| **Recombinant DNA** | |
| --- | --- |
| REAGENT or RESOURCE | SOURCE |
| pRK7-FLAG-STAT1 | This Study |
| pRK7-FLAG-STAT2 | This Study |
| pRK7-FLAG-STAT3 | This Study |
| pRK7-FLAG-STAT4 | This Study |
| pRK7-FLAG-STAT5A | This Study |
| pRK7-FLAG-STAT5B | This Study |
| pRK7-FLAG-STAT6 | This Study |
| pCMV-FLAG-STAT5A^S127A^ | This Study |
| pCMV-FLAG-STAT5A^S128A^ | This Study |
| pCMV-FLAG-STAT5A^S726A^ | This Study |
| pCMV-FLAG-STAT5A^Y694A^ | This Study |
| pCMV-FLAG-STAT5A^T757A^ | This Study |
| pCMV-FLAG-STAT5A^S780A^ | This Study |
| pCMV-FLAG-STAT5A^S127D^ | This Study |
| pCMV-FLAG-STAT5A^S128D^ | This Study |
| pCMV-FLAG-STAT5A^S726D^ | This Study |
| pCMV-FLAG-STAT5A^Y694D^ | This Study |
| pCMV-FLAG-STAT5A^T757D^ | This Study |
| pCMV-FLAG-STAT5A^S780D^ | This Study |
| pcDNA3.1b-PDP2-FLAG | This Study |
| pcDNA3.1b-ACO2-FLAG | This Study |
| pcDNA3.1b-FH-FLAG | This Study |
| pcDNA3.1b-OGDH-FLAG | This Study |
| pcDNA3.1b-IDH2-FLAG | This Study |
| pcDNA3.1b-ALDOA-FLAG | This Study |
| pcmv-mito-FLAG-STAT5A | This Study |
| pX459-STAT5A-KO | This Study |
| pCDH-STAT5A-4A | This Study |
| pCDH-STAT5A | This Study |
| pCDH-mito-STAT5A | This Study |
| pGEX-6P-1-STAT5A | This Study |

| **Primers or sequences** | |
| --- | --- |
| CRISPR/Cas9-mediated STAT5A gene editing primer-F  CACC G GATGGCACACTTCCAATG | This Study |
| CRISPR/Cas9-mediated STAT5A gene editing primer-R  AAACGTCCGCAAGCGTCCGGCCGC | This Study |
| CRISPR/Cas9-mediated HIF-1α gene editing primer-F  CACCGTTCTTTACTTCGCCGAGATC | This Study |
| CRISPR/Cas9-mediated HIF-1α gene editing primer-R  AAACGATCTCGGCGAAGTAAAGAAC | This Study |
| Q-PCR SOD1-F: TCATCAATTTCGAGCAGAAGG | This Study |
| Q-PCR SOD1-R: CAGGCCTTCAGTCAGTCCTTT | This Study |
| Q-PCR NQO1-F: CAGCTCACCGAGAGCCTAGT | This Study |
| Q-PCR NQO1-R: GAGTGAGCCAGTACGATCAGTG | This Study |
| Q-PCR HMOX1-F: GGGTGATAGAAGAGGCCAAGA | This Study |
| Q-PCR HMOX1-R: AGCTCCTGCAACTCCTCAAA | This Study |
| Q-PCR GCLC-F: ATGCCATGGGATTTGGAAT | This Study |
| Q-PCR GCLC-R: AGATATACTGCAGGCTTGGAATG | This Study |
| Q-PCR GAPDH-F: TGCACCACCAACTGCTTAGC | This Study |
| Q-PCR GAPDH-R: GGCATGGACTGTGGTCATGAG | This Study |
| Mito sequences: ATGTTCTTCTCCGCGGCGCTCCGGGCCCGGGCGGCTGGCCTCACCGCCCACTGGGGAAGACATGTAAGG | This Study |
